# X-ray Computed Tomography for Characterization of Expanded Polystyrene (EPS) Foam

**DOI:** 10.3390/ma12121944

**Published:** 2019-06-17

**Authors:** Redouane Meftah, Jeroen Van Stappen, Sylvain Berger, Gary Jacqus, Jean-Yves Laluet, Paul-Henri Guering, Luc Van Hoorebeke, Veerle Cnudde

**Affiliations:** 1 UGCT/PProGRess, Department of Geology, Ghent University, Krijgslaan 281/S8, 9000 Ghent, Belgium; J.F.Vanstappen@UU.nl (J.V.S.); Veerle.Cnudde@UGent.be (V.C.); 2 Saint-Gobain Research Paris, Department of Optics Metrology and Mathematics, 39 Quai Lucien Lefranc, 93300 Aubervilliers, France; Sylvain.Berger@saint-gobain.com (S.B.); Gary.Jacqus@saint-gobain.com (G.J.); Jean-Yves.Laluet@saint-gobain.com (J.-Y.L.); Paul-Henri.Guering@saint-gobain.com (P.-H.G.); 3 High Pressure and Temperature Laboratory, Department of Earth Sciences, Utrecht University, Budapestlaan 4, 3584CD Utrecht, The Netherlands; 4 UGCT/Radiation Physics, Department of Physics and Astronomy, Ghent University, Proeftuinstraat 86/N12, 9000 Ghent, Belgium; Luc.VanHoorebeke@UGent.be; 5 Department of Earth Sciences, Utrecht University, Princetonlaan 8A, 3584CD Utrecht, The Netherlands

**Keywords:** X-ray computed tomography, expanded polystyrene (EPS), contrasting agent, parametric study, sound absorption, Johnson–Champoux–Allard model

## Abstract

Expanded polystyrene (EPS) foam is widely used in building and construction applications for thermal and acoustic insulation. This material is nearly transparent for X-rays, making it difficult to characterize its pore structure in 3D with X-ray tomography. Because of this difficulty, the pore network is often not investigated and is, thus, poorly known. Since this network controls different physical properties, such as the sound absorption, it is crucial to understand its overall structure. In this manuscript, we show how to reveal the pore network of EPS foams through the combination of high resolution X-ray tomography (micro-CT) and saturation techniques. The foams were saturated with CsCl-brine, which acts as a contrasting agent in X-ray micro-CT imaging. This allowed us to separate the beads, making up the foam, from the pore network. Based on the 3D micro-CT results, we were able to assess a representative elementary volume for the polystyrene, which allows for calculating the acoustical parameters from the Johnson–Champoux–Allard (JCA) model, the pore and bead size distribution. The 3D data was also used as input to simulate sound absorption curves. The parametric study showed that an increase in the bead size influenced the sound absorption of the material. We showed that, by doubling the diameter of beads, the absorption coefficient was doubled in certain ranges of frequency.

## 1. Introduction

Expanded polystyrene foam (EPS) is a widely used modern material in automobile, building, and other industries [1,2]. Compared with non-foamed polystyrene plastics, EPS has a lower density, a lower thermal conductivity, and a higher load bearing strength per weight [3]. More importantly, the properties of polymer foams can be easily tuned by controlling the pore size, the relative density, the cell structure, and the use of additives [4,5,6]. 

EPS foam is a cellular material, used in various industrial sectors because of its remarkable properties. It is relatively stiff, easy to glue, lightweight, provides an excellent thermal insulation, and, especially, is much cheaper than other building materials. In addition, EPS is a polymer particularly unaffected by climatic conditions (heat, humidity, and ultraviolet and infrared radiation) and it resists very well to aging [7]. Besides this, tests carried out by Yamura et al. [8] showed the hydrophobic nature of EPS beads [9], making them completely impermeable to water. However, the use of EPS is an important environmental problem and has serious health consequences for humans [10]. It contains toxic substances, such as styrene and benzene [11], suspected neurotoxins and carcinogens, that could be harmful to humans when released.

EPS foam is typically composed of multiple beads which are assembled together. These beads are perfectly spherical and the cross section of a bead reveals a honeycomb structure and an envelope with several membranes. This explains, on the one hand, its extreme lightness and, on the other hand, its total impermeability to water. 

Due to their low density and low ability to attenuate X-rays, it is very difficult to fully characterize EPS beads and foams using X-ray micro-CT [12]. In this context, little research has been done on the characterization of the EPS itself by X-ray micro-tomography. In 2003 Michaels et al. [13] soaked EPS foam in olive oil to successfully observe the foam structure using X-ray micro-CT. In 2007, Bouvard et al. [14] reported on the study of EPS in concrete using X-ray micro-CT in combination with a fluorescent screen to distinguish the EPS. This data was used for further modelling to predict thermal and mechanical properties. Recently, to study the link between the microstructure and thermal variations, Maaroufi et al. [15], characterized the EPS in EPS lightweight concrete by X-ray tomography and considered that the EPS was included in the observed porosity.

In this context, we propose to use a saturation method for EPS material in combination with X-ray micro-CT to accurately measure intrinsic parameters and characterize the morphology of EPS material. Due to its low X-ray absorbance, the network of EPS is not well known and, in particular, the link between the microstructure and the acoustic properties of this material. The saturation techniques not only provide detailed and accurate knowledge of the microstructure, but, in combination with image processing and numerical simulations, demonstrate the effect of intrinsic parameters on acoustics absorption. Standard 3D analysis is then carried out to select a representative elementary volume and to obtain all macroscopic parameters (porosity, tortuosity, airflow resistivity, and thermal and viscous characteristic length), as well as the size distribution, needed for the understanding of the sound absorption of the materials using the micro-macro approach [16]. Particularly, the JCA model [17] is used in this study for the parametric study to investigate the role of some parameters in the sound absorption, which allows us to determine how the sound absorption of EPS foam can be improved.

## 2. Materials and Methods 

**Figure 1:**
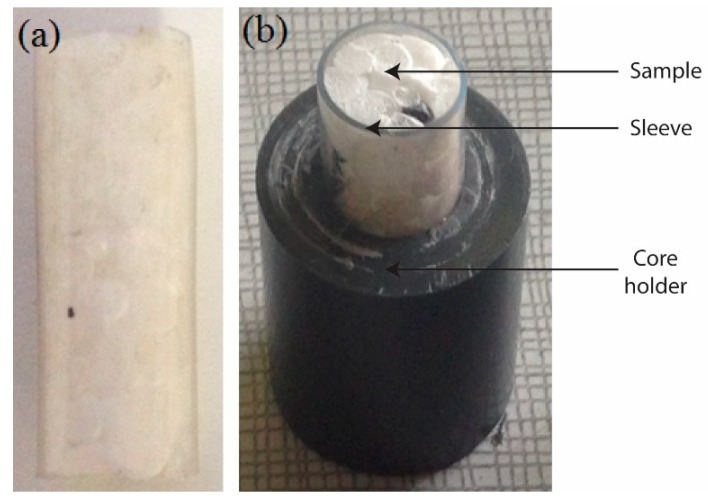
(**a**) Uncompressed sample with polytetrafluoroethylene (PTFE) sleeve; and (**b**) bottom part of the core holder (the top part is exactly the same as the bottom one and both parts are glued with epoxy glue).

Most of the time, EPS panels are bonded with gypsum or mortar boards in order to increase the thermal insulation or sound insulation of building elements. Therefore, two cylindrical samples of expanded polystyrene foam (diameter = 12 mm, length = 25 mm), a compressed and an uncompressed one, were used for this study. The samples were contained in a polytetrafluoroethylene (PTFE) sleeve, which was itself glued into a core holder made of poly(methyl methacrylate) (PMMA) (Figure 1). This was done to ensure that the injected fluids were pushed into the EPS sample instead of flowing around it.

**Figure 2:**
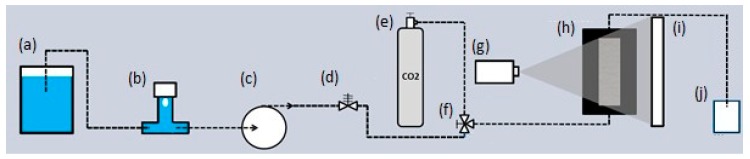
Schematic representation of the experimental setup with: (**a**) open fluid container, (**b**) bubble trap, (**c**) fluid pump, (**d**) pressure relief valve with an upper limit of 100 psi, (**e**) pressure-controlled CO_2_ gas, (**f**) three-way valve, (**g**) X-ray source of the EMCT, (**h**) sample in core holder, (**i**) detector of the EMCT, and (**j**) waste container.

In order to visualize the pore structure from the EPS beads and the EPS foam matrix, a dedicated fluid flow setup was built. This setup, with the controlling fluid flow lines, was installed on the Environmental Micro-CT (EMCT) scanner [18], the gantry-based micro-CT scanner of the Centre of X-ray Tomography of Ghent University (Ghent, Belgium). The complete setup is schematically represented in Figure 2. This setup is based on the fluid flow setup described in Van Stappen et al. [19], with less elements. It starts from an open fluid container to the constant flow pump. In between, a bubble trap is placed to avoid air bubbles in the flow line. After the fluid pump, the flow line begins with a pressure relief valve, which limits the pressure up to 100 psi, and then passes a three-way control valve, which allows for the switch between CO_2_ and the flow of liquid from the open fluid container. Then, the fluid flow line goes to the bottom part of the sample in the core holder and leaves the fluid flow cell at the top, to reach an open fluid drain.

To clearly distinguish the EPS matrix, the samples were first fully saturated with CO_2_ for 10 min at a pressure of 1.5 bar in order to remove all air. Afterwards, the three-way valve was switched to pump a 10 wt.% solution of cesium chloride (CsCl), which acted as a contrast agent in the micro-CT scans, with a maximum flow rate of 5 µL/s. In contact with the brine, the CO_2_ dissolved and the samples were fully saturated. Micro-CT scans were taken before and after fluid saturation. Table 1 compiles the scanning parameters for the micro-CT scans conducted in these experiments.

The micro-CT images were reconstructed with the Octopus Reconstruction software (version 8.9.4, Tescan-XRE, Ghent, Belgium) [20], after which 3D image analysis was performed with Avizo (version 2019.1, Thermo Fisher Scientific, Waltham, MA, USA) and the parametric study was done with ScalingCell (5.2.1 version, Matelys, Vaulx-en-Velin, France) [16]. 

In order to predict the acoustic absorption of the EPS, the JCA model [17] was used. This model links five intrinsic parameters: porosity φ (-), tortuosity α_∞_ (-), air flow resistivity σ (N·s·m^−4^), thermal characteristic length Λ (m), and viscous characteristic length Λ’ (m) to the acoustic absorption.

## 3. Results and Discussion

Obtaining a 3D volume representation of the material with the saturation methods allows the identification of the different phases. Additionally, it permits the evaluation of the intrinsic parameters of the EPS samples: micro-CT scanning of the uncompressed and compressed samples allows for the evaluation of the open porosity values, thus helping in the determination of the representative elementary volume (REV) and the volume fractions of each phase (pores and EPS beads). After that, bead and pore size distributions (PSD and BSD, respectively), in addition to the evolution of the porosity values in the Z-direction, were extracted. Then, the five intrinsic parameters and the characteristic bead size were determined to perform parametric studies by modeling.

### 3.1. Determination of REV, Porosity, PSD, and BSD

**Figure 3:**
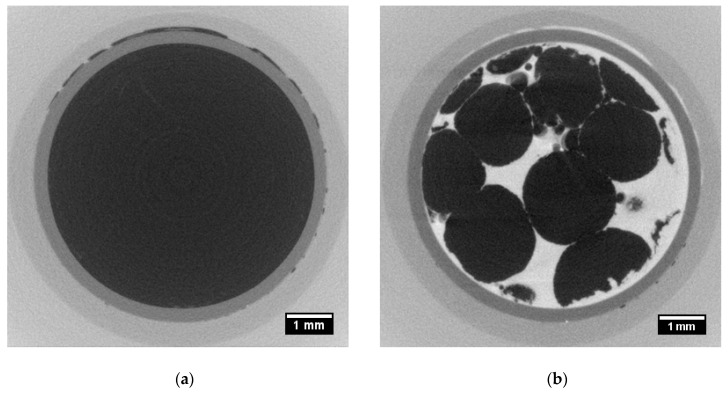
Tomographic slice of the uncompressed sample (**a**) before saturation and (**b**) after saturation.

Figure 3 illustrates the obtained micro-CT data of the uncompressed EPS (a) before and (b) after saturation. EPS is a very low density material, between 10 kg/m^3^ and 30 kg/m^3^ [9], and therefore attenuates X-rays very poorly, as illustrated in Figure 3a. At a source voltage of 50 kV and output power of 15 W, nearly all X-rays passed through the material and only the core holder and the sleeve were visible (Figure 3a). Polystyrene beads and pores share the same grey level and are undistinguishable. Figure 3b shows the fully saturated sample with CsCl-brine.

**Figure 4:**
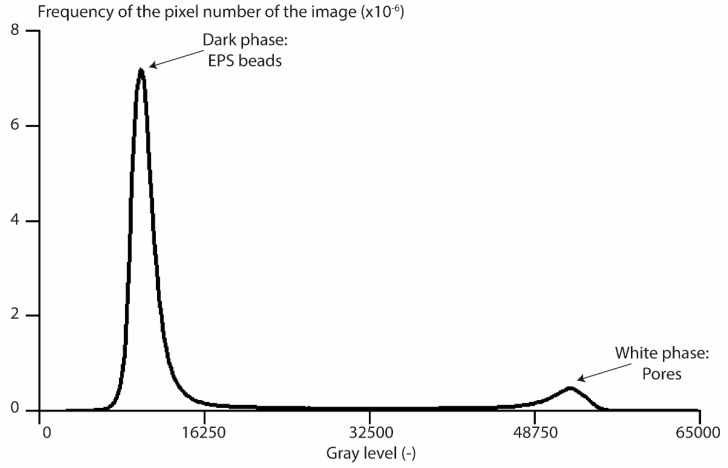
Gray level histogram of the uncompressed sample extracted from the 3D reconstructed tomographic volume.

Two phases can clearly be observed and confirmed from the grey level histogram (Figure 4), obtained after image processing of the 3D tomographic images. The graph shows two peaks equivalent to the two phases. The first one corresponds to the dark phase, which represents the EPS beads, and the second one corresponds to the white phase, which represents the CsCl-brine within the pore network.

**Figure 5:**
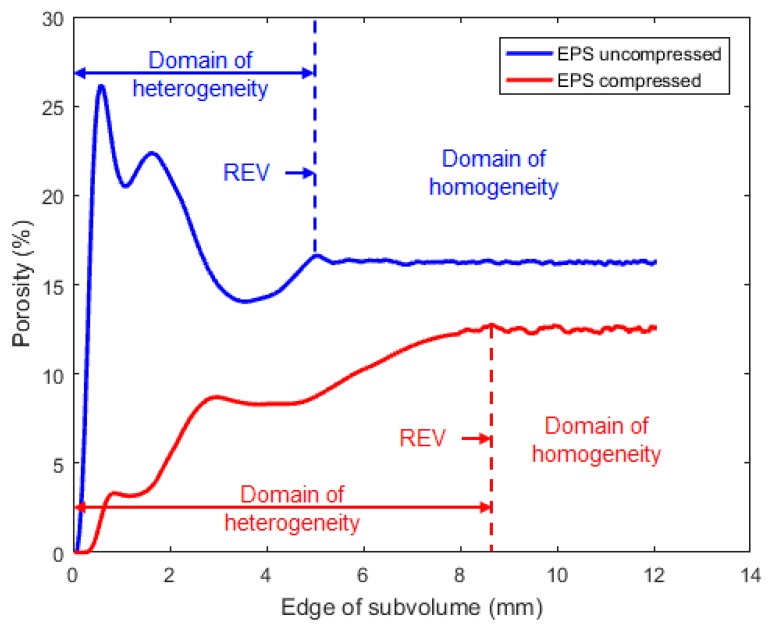
Determination of the representative elementary volume (REV) based on porosity measurements.

Based on this histogram, it is very easy to distinguish the EPS beads and the porosity with the saturation method. In order to determine the REV, the porosity was measured for cubic sub-volumes, increasing in size, located within the center of the samples, and this was computed for the uncompressed and compressed sample, as shown in Figure 5, in terms of the edge length of sub volume.

The results indicated that, for small volumes, there are important variations in porosity because of small scale random fluctuations associated with pore scale heterogeneity. These variations decreased as the sub-volume increased. The domain of homogeneity was reached for an edge value of 5.1 mm for the uncompressed sample and 8.9 mm for the compressed sample, which correspond to a volume of 132 mm^3^ and 705 mm^3^, respectively. In the compressed sample there was a greater influence of the polystyrene particles than in the uncompressed one. This explains the difference in REV. Moreover, because of the compression process, more “small” pores were formed in the compressed sample, which created more small-scale heterogeneity. The measured mean value of porosity was 16.1% and 11.6%, for the uncompressed and compressed sample, respectively. These values are based on the segmentation of the CsCl-brine in the micro-CT images. Experimental measurements were also carried out using nitrogen porosimetry, which gave 16.0% and 11.1% for the uncompressed and compressed sample, respectively. The measurements based on micro-CT images are thus in total agreement with the porosimetry ones.

**Figure 6:**
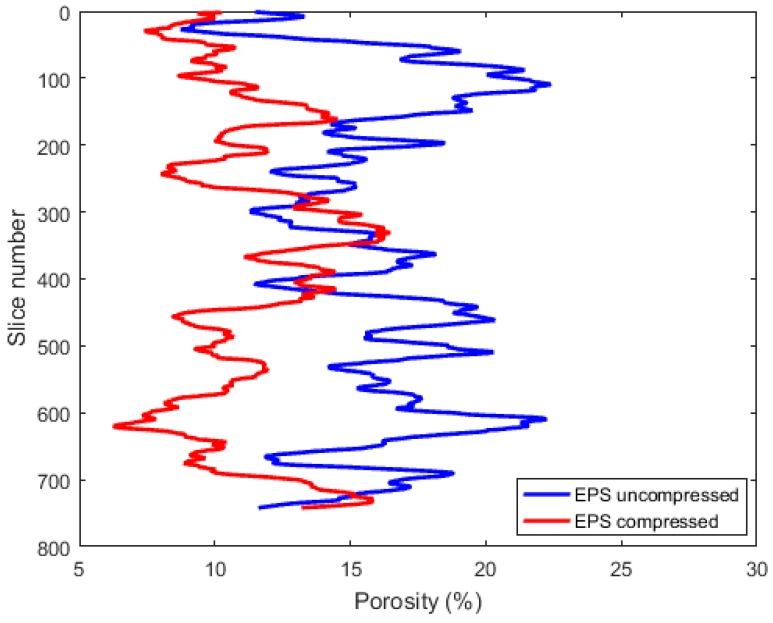
Porosity profile along the vertical direction; one slice had a thickness of 20 µm.

The porosity distribution in the vertical Z-direction is presented in Figure 6, which was obtained by calculating the average 2D porosity in each horizontal slice through the micro-CT data. The curves show an arbitrary distribution of the porosity in the vertical direction. Large variations between successive images can also be observed, which indicate many discontinuities in general. For the uncompressed sample, the porosity values ranged from 8.7% to 22.4%, and, for the compressed sample, the porosity values ranged from 6.3% to 16.5%.

**Figure 7:**
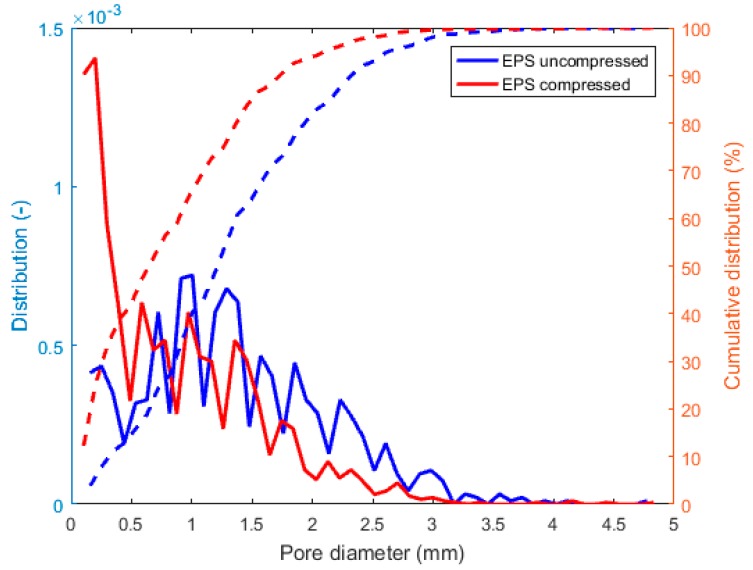
Pore size distribution and its cumulative distribution of the uncompressed and compressed specimens.

The pore size distribution of the two samples, normalized by number and obtained using the Avizo software suite, is shown in Figure 7. Pores with sizes smaller than 60 µm (3 voxels) were not taken into account. According to Figure 7, small (micron-sized) and big (millimeter sized) diameter sizes can be observed. The cumulative distribution functions were used to identify the likely range of the pore size and showed that 50% of the pores were smaller than 1.25 mm in diameter for the uncompressed sample, while, 50% of the pores were smaller than 0.68 mm in diameter for the compressed sample. It showed that the uncompressed sample had around 30% more pores bigger than 1 mm when compared to the compressed sample. The mean pore diameters were 0.84 mm and 1.35 mm for the compressed and uncompressed sample, respectively.

**Figure 8:**
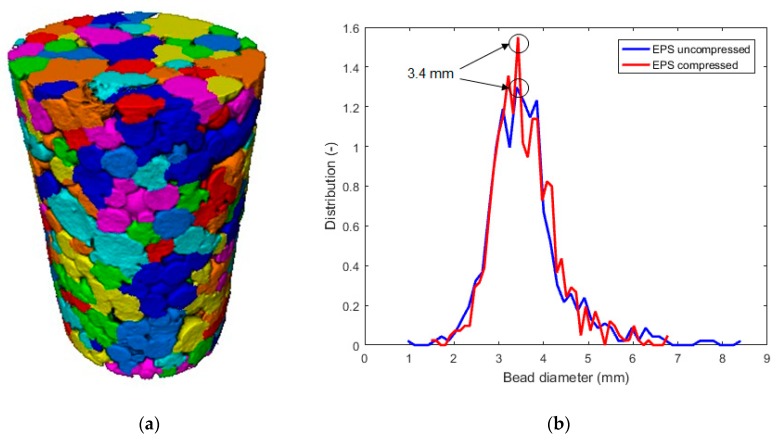
(**a**) Individualization of the different EPS beads, visualized by artificial color code in the compressed sample, and (**b**) bead size distribution for uncompressed and compressed EPS.

**Figure 9:**
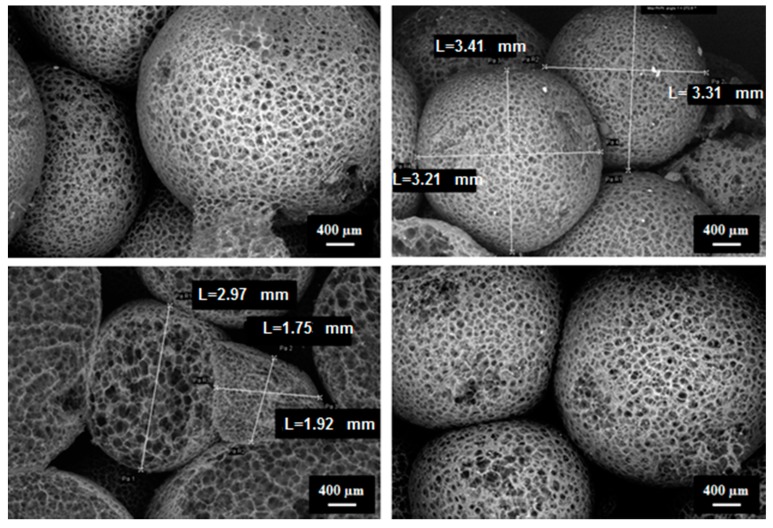
SEM images of EPS samples carried out at Saint-Gobain Research Paris.

Based on the micro-CT data, it is possible to digitally separate the EPS beads (Figure 8a) in Avizo for the quantification of their size. Figure 8b shows the resulting bead size distribution of the two specimens. Both samples had the same characteristic bead size: 3.4 mm. This characteristic size is in agreement with the observations done using scanning electron microscopy (SEM) in Figure 9.

### 3.2. Determination of Tortuosity and Characteristics Lengths

The tortuosity of a path, formed by the centroids on each slice along the z-axis of a 3D image, was also determined. Tortuosity is defined as the ratio between the length of the path and the distance between its ends along the z-axis. In our case, the distance between the ends of the path is given by the number of slices along the z-axis. Avizo first computes the centroid of segmented pores for each horizontal slice of the image. Then it computes the path length through the centroids and divides it by the number of slices along the z-axis. With the XLab extension within Avizo, the absolute permeability of the samples can be computed and be related to the air flow resistivity, which is an important parameter for understanding the acoustical absorption of the material. The relationship between the absolute permeability K and the air flow resistivity σ is given by the following equation (Equation (1)):

(1) $$\sigma ={µ}_{air}/K_{ZZ},$$

where $\mu_{air}$ is the dynamic viscosity of air (1.48 × 10^−5^ Pa·s at 20 °C) and $K_{zz}$ the transverse absolute permeability in m^2^. The viscous and thermal characteristic lengths were also determined. Both were computed with ScalingCell. The thermal characteristic length is a geometrical parameter defined as the hydraulic radii of pores. The viscous characteristic length is computed in the high frequency limit assuming an inviscid fluid [16]. The intrinsic parameters computed for both samples are summarized in Table 2.

In general, the uncompressed sample was a bit more tortuous than the compressed sample. The results showed that the compressed sample was more resistive than the uncompressed sample, mainly because of a lower porosity. The characteristics lengths were higher in the uncompressed sample.

**Figure 10:**
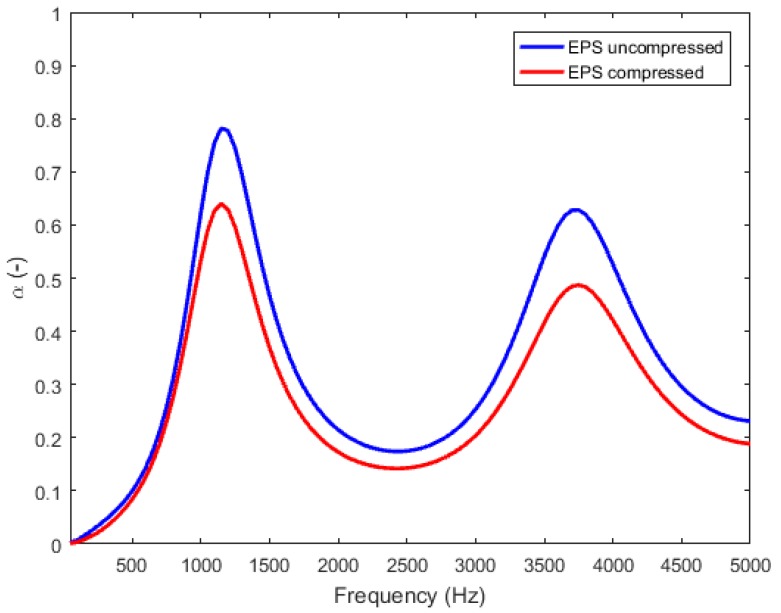
Normal sound absorption spectra of the uncompressed and compressed EPS foam modelled using ScalingCell.

These parameters allowed us to predict the normal sound absorption curves of the two samples, assuming a rigid backing (Figure 10). It shows two absorption peaks for the two samples (around 1100 Hz and 3750 Hz), which are related to the thickness resonances of the compression wave inside the porous material. Since the propagation regime is mainly inertial for EPS foams, the position of the resonance has the following expression (Equation (2)):

(2) $$f=\frac{c}{4h}≅\frac{c_{air}}{4h\sqrt{\alpha_{\infty }}}.$$

Additionally, it can be observed that the absorption for the uncompressed sample is higher than the one for the compressed sample.

### 3.3. Parametric Studies by Modelling

**Figure 11:**
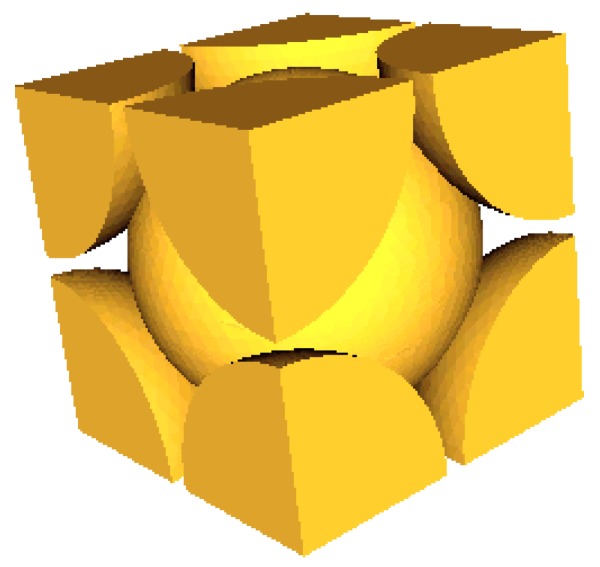
EPS cell with a face-centered cubic lattice.

Based on the 3D characterization of the polystyrene samples, it was possible to perform a parametric study using ScalingCell in order to understand the influence of microstructural parameters on material performance. In ScalingCell, an EPS cell with a face-centered cubic lattice (Figure 11) was created. For this parametric study, the focus was on the parameters of the uncompressed sample. 

**Figure 12:**
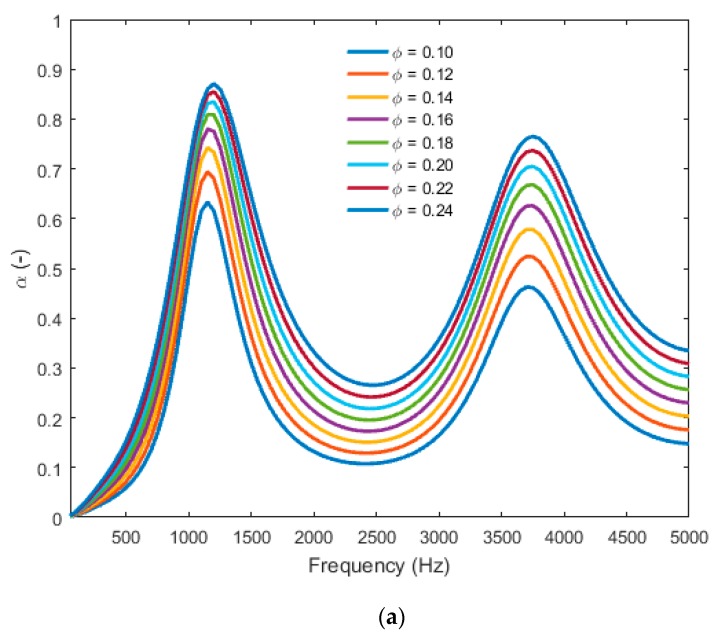

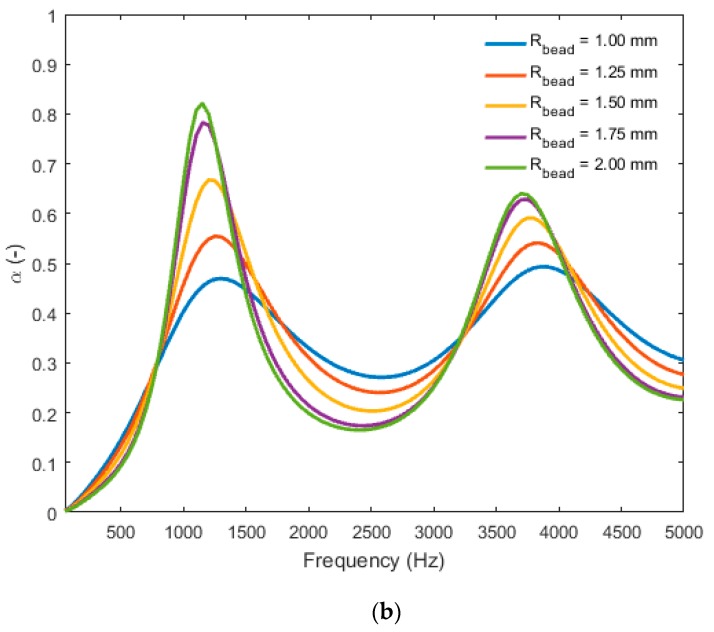
(**a**) Absorption curves at normal incidence with multiple bead sizes and porosity fixed at 16.1% and (**b**) absorption curves at normal incidence with multiple porosities and bead size fixed at 1.7 mm.

The absorption peaks were maximized at a fixed porosity for a maximum bead size (Figure 12a). This is equivalent to a minimum value of airflow resistivity. Likewise, at a fixed bead size, an increase in porosity gave a higher absorption peak (Figure 12b). With this parametric study, two ways to improve the acoustic absorption of the material were found: (1) decreasing the resistivity and (2) increasing the porosity of the EPS foam. At a fixed porosity it would, therefore, be sufficient to increase the radius of the polystyrene grains, and, at a fixed grain size, to increase the porosity. Also, care must be taken to respect the specifications of the mechanical properties, which lead most of the time to compromises, especially when the Young’s modulus [21] and Poisson’s ratio [22] decrease when the porosity increases. Notably, this could be carried out by meshing the 3D cell and performing numerical simulations of the mechanical parameters using finite element methods (FEM) [23] or by assessing some parameters, such as the roughness, with confocal microscopy [24].

## 4. Conclusions

In this work, X-ray micro-CT was used for the morphological characterization of two samples of expanded polystyrene foam. In addition, based on the packing observed in the micro-CT images, a parametric study was performed in order to understand which parameters could increase the acoustic absorption.

By saturating the EPS with a contrasting agent, the different phases of the material (porosity versus polystyrene beads) could be clearly distinguished.

The 3D images allowed us to compute the five parameters of the JCA model for both samples: porosity, tortuosity, airflow resistivity, and characteristics lengths. We could then assess the representative elementary volume as 8.9 × 8.9 × 8.9 mm^3^ for the compressed sample and 5.1 × 5.1 × 5.1 mm^3^ for the uncompressed one. We also determined the pore size distribution of the material, which showed its complex microstructural heterogeneity. 

The bead size distribution was also determined and we found the same characteristic polystyrene bead size for both samples. This corresponded to a bead diameter of 3.4 mm and was in agreement with the observation made with SEM. 

Finally, a parametric study was done and helped explain the influence of some parameters in the absorption of the material. We found that an increase in the size of the beads will help in increasing the absorption. An increase in bead size of 100% will cause a double increase in the peak absorption.

## Figures and Tables

**Table 1:** Scanning parameters of the experiments.

Micro-CT System	Source Voltage (kV)	Output POWER (W)	# Projections Per Scan	Exposure Time (ms)	Frame Averages	Scan Time (min)	Voxel Size (µm)
EMCT	50	15	1441	175	4	17	20

**Table 2:** Johnson–Champoux–Allard parameters calculated from the images.

Sample	Porosity (%)	Tortuosity (-)	Airflow Resistivity (N·s·m^−4^)	Viscous Characteristic Length (µm)	Thermal Characteristic Length (µm)
**Uncompressed**	16.1	1.61	25250	183	287
**Compressed**	11.6	1.55	32700	172	211
